# Time-lapse imaging of *C. elegans* egg-laying with high spatiotemporal resolution

**DOI:** 10.17912/micropub.biology.001914

**Published:** 2025-11-20

**Authors:** Siddharthan Balachandar Thendral, Nicole Roos, Johnny Vertiz, Anthony S. Wokasch, Paul Maier, David R. Sherwood

**Affiliations:** 1 Department of Biology, Duke University, Durham, North Carolina, United States; 2 Department of Biology, Emory University, Atlanta, Georgia, United States; 3 Department of Developmental and Cell Biology, University of California, Irvine, Irvine, California, United States; 4 Department of Cell and Developmental Biology, Vanderbilt University, Nashville, Tennessee, United States; 5 Multiscale biology, University of Göttingen, Göttingen, Lower Saxony, Germany

## Abstract

Egg-laying behavior in *C. elegans* is driven by a simple neural circuit comprising motor neurons, vulva muscles, and neuroendocrine cells. Most protocols designed to capture this behavior rely on imaging freely moving animals at low magnifications, thus limiting subcellular-level understanding of the underlying neuromuscular circuit. Here, we present a protocol to immobilize *C. elegans* while maintaining egg-laying activity, enabling high spatiotemporal-resolution imaging of this behavior.

**Figure 1. A protocol for time-lapse imaging of C. elegans egg-laying with high spatiotemporal resolution on a glass slide:**
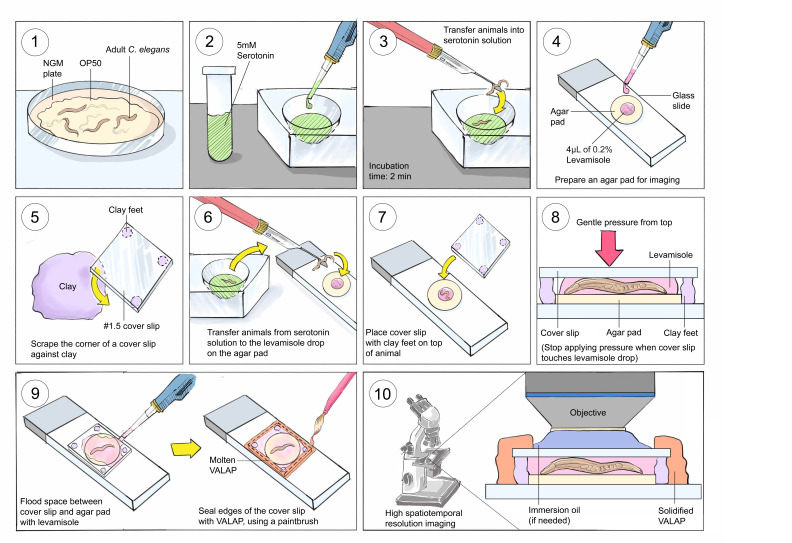
Panels numbered 1 to 10 correspond to steps 1 to 10 of the imaging protocol in the Description section. (Note: Serotonin and levamisole solutions are colorless. Colors were chosen solely for visual clarity.)

## Description

Egg-laying behavior of *C. elegans* is well characterized at both the genetic and neuromuscular levels. However, the rapid dynamics of this behavior (2-4 seconds per event), together with limitations in current imaging techniques, make it challenging to visualize at high spatial and temporal resolution. While anesthetics such as sodium azide and tricaine are used to immobilize worms and allow live-imaging, they also inhibit egg-laying behavior. The M9 salt solution, commonly used as an imaging buffer for worms, is also a potent inhibitor of egg-laying (Schafer 2006). Moreover, in standard confocal imaging, *C. elegans* are mounted on glass slides with overlying coverslips that exert physical pressure. The pressure on the uterine tissue triggers egg expulsion, preventing imaging of egg-laying. Because of these limitations, studies documenting *C. elegans* egg-laying have used experimental approaches where freely moving animals are imaged at low magnifications (20x) (Yan et al. 2025; Collins et al. 2016). Although this approach enables collection and correlation of animal locomotion data alongside egg-laying behavior, it does not allow the higher resolutions and magnifications (40x, 60x or 100x) achievable with slide-based confocal imaging of immobilized *C. elegans* . In this study, we provide a protocol for time-lapse imaging of egg-laying behavior in *C. elegans* immobilized on a glass slide using confocal microscopy. The improved resolution achieved through this protocol enables high resolution tissue and even sub-cellular level investigations of egg-laying behavior.

First, to overcome limitations imposed by anesthetics like sodium azide, we used levamisole—a nicotinic acetylcholine agonist that immobilizes *C. elegans* while stimulating egg-laying (Kim et al. 2001; Manjarrez and Mailler 2020). To further promote egg-laying behavior, we treated worms with serotonin prior to levamisole-mediated immobilization. Serotonin is a neuromodulator that facilitates the transition from an inactive to an active state of egg-laying in *C. elegans* (Waggoner et al. 1998). Because hypertonic salt solutions such as M9 buffer inhibit egg-laying (Schafer 2006), we prepared levamisole and serotonin solutions with autoclaved water instead of standard M9 buffer. Next, to prevent coverslip placement on top of animals from inducing immediate egg-laying, we placed small clay feet at each corner of the coverslip. This elevated the coverslips and then facilitated adjustment, such that the coverslip was made to be in contact with the worm cuticle without exerting pressure on the worm. After these modifications to the standard confocal imaging protocol, we were able to consistently live-image egg-laying behavior at high magnifications (40x and 60x) using spinning-disk confocal microscopes. With this protocol, we performed both brightfield imaging (Video 1) and confocal fluorescence imaging (Video 2) of egg-laying behavior.

Here, we present the protocol for inducing and live-imaging egg-laying (Fig 1):

1. Grow the *C. elegans* strain of choice in petri dishes containing ~250 µL *E. coli* strain OP50 spread over ~8 mL of solidified nematode growth medium (NGM). Transfer animals to fresh OP50 plates every three days to prevent starvation. Day 1 adult hermaphrodites (first day of reproductive adulthood) are most optimal for observing egg-laying behavior.

2. To induce egg-laying, treat *C. elegans* with serotonin. Prepare 5mM stock solution of serotonin in autoclaved deionized water and add 4-5 µL of this solution to a well of a multi-well plate.

3. Transfer 3-5 day 1 adult hermaphrodites onto the drop of serotonin using a heat-sterilized platinum pick. Incubate for 2 minutes.

4. Prepare an agar pad on a glass slide using ~100 µL of molten 5% noble agar. Add 4 µL of 0.2% levamisole diluted in diH _2_ O to the center of the agar pad. Do not use M9 buffer, as salt solutions inhibit egg-laying.

5. Create clay feet on a #1.5 coverslip by gently scraping each corner against clay. Clay feet with height slightly more than the working distance for the specific objective and microscope (~0.25mm to 0.40mm) is recommended. Ensure even amounts of clay at all four corners of the coverslip to maintain a horizontally level orientation during imaging.

6. After 2 minutes of incubation in serotonin, promptly transfer the worms onto the levamisole drop on the agar pad.

7. Gently place the coverslip with clay feet at its corners onto the worms in solution, on the agar pad. The clay feet create sufficient space between the coverslip and worms, preventing increased pressure on animals. Placement of a coverslip without clay feet typically causes immediate egg expulsion.

8. Apply gentle pressure to the corners of the coverslip, sufficient only to bring the levamisole drop into contact with the coverslip surface.

9. Using a micropipette, fill any remaining air pockets beneath the coverslip with 0.2% levamisole solution, and quickly seal the coverslip edges with molten VALAP (a 1:1:1 mixture of Vaseline, lanolin, and paraffin). VALAP prevents drying of the agar pad and allows time-lapse imaging for relatively long timeframes, increasing chances of capturing egg-laying (up to 2h).

10. Perform time-lapse imaging of the vulva region using a fluorescent marker of choice, or under DIC with a suitable microscope. Use short time intervals between image acquisitions to capture the various stages of egg-laying.

Finally, we include a model overlayed on images captured using our protocol, showing the course of events that constitute egg-laying behavior (Video 2) to provide an overview of the *C. elegans* egg-laying circuit.

## Methods

**Culturing *C. elegans* strains:**

*C. elegans* strains used include the Bristol N2 wild-type strain and LP399 ( *cp151 [mNG::3xFlag:: rap-1* ]) (Dickinson et al. 2015). Both strains were maintained at 25°C on nematode growth medium plates containing *E. coli* strain OP50 .

**Microscopy:**

Time-lapse images of the LP399 strain were acquired using a Nikon CSU-W1 spinning disk confocal microscope. Brightfield images of wild-type animals was captured using an ECHO spinning disk confocal. Imaging was performed with 40x Plan Apochromat air and water immersion objectives, using excitation from a 488 nm laser line. Worms were mounted on 5% noble agar pads under #1.5 coverslips. Video 1 was recorded at ~9 fps, and Video 2 at ~2.27 fps. Both videos were single-slice confocal image acquisitions. For 3D z-stack imaging, frame rates ranging from 2-20 fps are achievable with this protocol, provided the tagged protein of interest is sufficiently bright to allow short exposure times, which is important for rapid acquisition of images. Images were processed using Fiji 2.0 (v1.54r).

Adobe illustrator (v29.8.3), Procreate (v5.2.9), and Adobe Premier Pro (v25.5.0) were used for preparation of this manuscript.

## Reagents

*C. elegans* strains: Bristol N2 wild-type strain and LP399 ( *cp151 [mNG::3xFlag:: rap-1* ])

## Data Availability

Description: Time-lapse brightfield confocal imaging of C. elegans egg-laying:Egg-laying behavior in a day-1 adult hermaphrodite is shown . Resource Type: Audiovisual. DOI: https://doi.org/10.22002/2q5jw-tm927 Description: Time-lapse confocal fluorescence imaging of C. elegans egg-laying: In a day-1 adult hermaphrodite, plasma membranes of embryos and the cells constituting the egg-laying apparatus are visualized using mNG::RAP-1 (Dickinson et al. 2015). Egg-laying behavior is captured . Resource Type: Audiovisual. DOI: https://doi.org/10.22002/4xgvx-q8249
